# Three-step docking by WIPI2, ATG16L1, and ATG3 delivers LC3 to the phagophore

**DOI:** 10.1126/sciadv.adj8027

**Published:** 2024-02-07

**Authors:** Shanlin Rao, Marvin Skulsuppaisarn, Lisa M. Strong, Xuefeng Ren, Michael Lazarou, James H. Hurley, Gerhard Hummer

**Affiliations:** ^1^ Department of Theoretical Biophysics, Max Planck Institute of Biophysics, Frankfurt am Main, Germany.; ^2^ Aligning Science Across Parkinson’s (ASAP) Collaborative Research Network, Chevy Chase, MD 20815, USA.; ^3^ Walter and Eliza Hall Institute of Medical Research, Melbourne, Victoria, Australia.; ^4^ Department of Biochemistry and Molecular Biology, Biomedicine Discovery Institute, Monash University, Melbourne, Victoria, Australia.; ^5^ Department of Molecular and Cell Biology, University of California, Berkeley, Berkeley, CA 94720, USA.; ^6^ California Institute for Quantitative Biosciences, University of California, Berkeley, Berkeley, CA 94720, USA.; ^7^ Department of Medical Biology, University of Melbourne, Melbourne, Victoria, Australia.; ^8^ Helen Wills Neuroscience Institute, University of California, Berkeley, Berkeley, CA 94720, USA.; ^9^ Institute of Biophysics, Goethe University Frankfurt, 60438 Frankfurt am Main, Germany.

## Abstract

The covalent attachment of ubiquitin-like LC3 proteins (microtubule-associated proteins 1A/1B light chain 3) prepares the autophagic membrane for cargo recruitment. We resolve key steps in LC3 lipidation by combining molecular dynamics simulations and experiments in vitro and in cellulo. We show how the E3-like ligaseautophagy-related 12 (ATG12)–ATG5-ATG16L1 in complex with the E2-like conjugase ATG3 docks LC3 onto the membrane in three steps by (i) the phosphatidylinositol 3-phosphate effector protein WD repeat domain phosphoinositide-interacting protein 2 (WIPI2), (ii) helix α2 of ATG16L1, and (iii) a membrane-interacting surface of ATG3. Phosphatidylethanolamine (PE) lipids concentrate in a region around the thioester bond between ATG3 and LC3, highlighting residues with a possible role in the catalytic transfer of LC3 to PE, including two conserved histidines. In a near-complete pathway from the initial membrane recruitment to the LC3 lipidation reaction, the three-step targeting of the ATG12–ATG5-ATG16L1 machinery establishes a high level of regulatory control.

## Teaser

Three distinct membrane-docking steps of the ATG12–ATG5-ATG16L1-ATG3–LC3 complex control LC3 lipidation at the phagophore.

## INTRODUCTION

Eukaryotic cells use autophagy for the wholesale degradation of bulk cytosol and bulky substrates, including intracellular pathogens, protein aggregates, and mitochondria (*1*). Autophagy of the latter is referred to as mitophagy (*2*, *3*). Defects in mitophagy downstream of the E3 ubiquitin (Ub) ligase Parkin and the Ub kinase PTEN-induced kinase 1 (PINK1) are implicated in familial Parkinson’s disease (*4*). Autophagy is critical for cell homeostasis across a vast range of physiological conditions, and its defects contribute to essentially all the major late-onset neurodegenerative diseases, cancer, and other diseases (*5*). The covalent conjugation of the Ub-like autophagy-related 8 (ATG8) proteins to the membrane lipid phosphatidylethanolamine (PE) is a hallmark of autophagy (*6*). Atg8 is the sole and founding member of this family in yeast, and it has six orthologs in humans, LC3A/B/C, GABARAP, and GABARAPL1/2 (*7*). ATG8 family proteins bind to short motifs known in humans as LC3-interacting regions (LIRs). LIR motifs are found throughout the machinery of autophagy, where their interactions facilitate cargo sequestration in selective autophagy (*8*), autophagosome-lysosome fusion, and autophagic membrane breakdown (*9*, *10*), and, indeed, have some role in most steps in autophagy.

ATG8s are conjugated to membrane PE through a pathway that has both analogies and differences with protein ubiquitylation. Ub and Ub-like proteins are conjugated to proteins, usually via the ε-amino group of Lys residues, by the sequential action of E1, E2, and E3 enzymes (*11*, *12*). The chemistry and structural biology of protein ubiquitylation, and the related Neddylation, SUMOylation, and similar pathways, have been elucidated in great detail (*11*, *12*). ATG8 conjugation begins with the action of the E1-like ATG7 and the E2-like ATG3 enzymes (*6*). These enzymes have the same overall fold and active-site cysteine residue as their cognate Ub E1 and E2 enzymes (*13*), as well as unique modifications that facilitate their mutual interaction (*14*) and the interaction of ATG3 with membranes (*15*). Purified ATG3 can carry out ATG8ylation on highly curved liposomes in vitro (*15*) in the absence of its cognate E3, but in vivo (*16*) and in a giant unilamellar vesicle reconstitution system (*17*, *18*), the downstream E3 complex components are essential.

The autophagic counterpart of the Ub E3 is the ATG12–ATG5-ATG16L1 complex (*19*), which is structurally and evolutionarily unrelated to any of its functional equivalents in ubiquitylation. The ATG12-ATG5 unit is itself covalently bonded through an ATG10-dependent reaction (*20*). ATG12-ATG5 binding allosterically activates ATG3 by increasing the exposure and reactivity of its Cys^264^-linked ATG8 thioester for transfer to PE (*14*, *21*). The ATG16L1 portion of the complex is responsible for delivery and positioning on the membrane (*19*). ATG16L1 is itself delivered to membranes by the β-propeller protein WD repeat domain phosphoinositide-interacting protein 2 (WIPI2) (*18*, *22*). WIPI2 (and other WIPIs) are recruited to membranes early in autophagy induction by the lipid phosphatidylinositol 3-phosphate [PI(3)P] (*23*), which is generated by the class III PI 3-kinase complex I (PI3KC3-C1) early in autophagy initiation (*24*).

The problem of how the chemistry and structural biology of a protein ubiquitylation-like system is adapted to act on a membrane substrate has been one of the major open questions in the mechanistic biochemistry of autophagy. A number of pieces of the puzzle have come together in recent years. The structural basis of the assembly of a fragment of ATG3 with the ATG12–ATG5-ATG16L1 unit was worked out for the human proteins (*21*). ATG16L1 contains an amphipathic helix α2, adjacent to its ATG5 binding site, which is strongly sensitive to membrane curvature (*25*) and essential for promotion of LC3 lipidation in liposomes and in cells (*26*). It is puzzling that ATG16L1 α2 is so important for catalysis, given that WIPI2 is capable of recruiting ATG16L1 to membranes through its WIPI2-interacting region (W2IR) (*18*, *22*). The structural basis for human ATG16L1 recruitment by WIPI2 has also been worked out (*18*, *27*). The ATG12-ATG5 and WIPI2-binding regions of ATG16L1 are separated by a coiled coil with >100 amino acids. The resulting extended shape and its flexibility challenge experimental structure determination of the full membrane-bound WIPI2-ATG12–ATG5-ATG16L1-ATG3 system. Here, we approached the problem beginning with large-scale all-atom simulations of the WIPI2-ATG12–ATG5-ATG16L1-ATG3 on lipid membrane. Predictions from the simulations were verified experimentally in vitro and in cellulo. In this way, we connect structural and biochemical information into a near-complete view of the lipidation pathway.

## RESULTS

### Docking step 1: WIPI2 recruits ATG12–ATG5-ATG16L1 loaded with ATG3-LC3 to phagophore

**Fig. 1.**
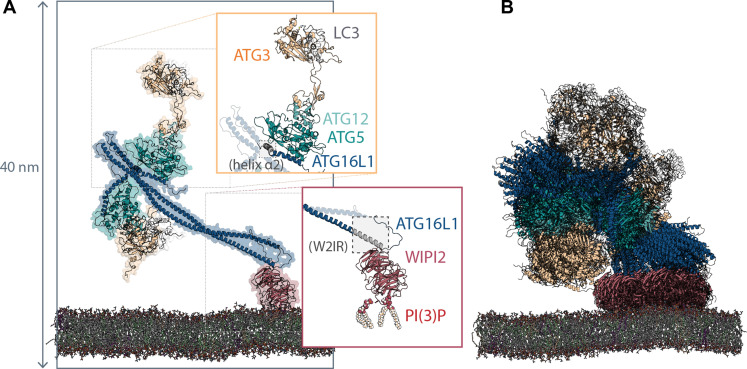
Structure and dynamics of the membrane-recruited ATG12–ATG5-ATG16L1-WIPI2 complex loaded with ATG3-LC3. (**A**) Ribbon and (semitransparent) surface representation of the full LC3 lipidation machinery bound to a membrane mimicking the phagophore lipid composition, upon equilibration and atomistic MD simulation. The ATG16L1 N-terminal helix α2 and the W2IR are highlighted in gray. (**B**) Dynamics of the assembly, illustrated with a superimposition of conformations sampled at 50-ns intervals during the final 500 ns of one 1-μs simulation trajectory. Flexibility of interdomain loops allows the ATG3-LC3 conjugate (yellow/white) to explore the region of space above the membrane to which the ATG12–ATG5-ATG16L1 is anchored.

As a key first step in targeting the lipidation machinery to the phagophore membrane, we concentrated on the WIPI2-mediated membrane interaction of ATG12–ATG5-ATG16L1. The central homodimer-forming coiled-coil domain (residues 78 to 230) of the human ATG16L1 protein is predicted (*28*) to form a continuous stretch of α-helical coiled coils spanning the major part of the domain (~115 amino acids from the N-terminal side), allowing reconstruction of the dimeric ATG16L1 structure by fitting geometric parameters (*29*) based on Crick’s equations (fig. S1A) (*30*). The resulting coiled-coil structure is in excellent agreement with crystal structures (*31*, *32*) of the mouse ortholog in which an overlapping region of the coiled-coil domain has been resolved (fig. S1A), providing validation for our ATG16L1 model. Using AlphaFold (*33*, *34*), we also obtained a structural model of the E2-like ATG3 conjugase loaded with LC3 (fig. S1B). The predicted ATG3-LC3 complex adopts a conformation compatible both with binding to the E1-like ATG7 homodimer in the preceding step and with formation of a thioester bond between the catalytic Cys^264^ side chain of ATG3 and the C-terminal Gly^120^ of LC3 to yield the E2-substrate conjugate (fig. S1B). The core of the human ATG3 structure is architecturally similar to the yeast and *Arabidopsis* Atg3 proteins, as has been previously reported (*35*), with an intrinsically disordered region (*36*) forming a ~100-residue loop that contains the ATG12-binding sequence (*21*) (Fig. 1A) as well as a region predicted to participate in β sheet formation in the presence of LC3 (fig. S1C). The AlphaFold-predicted intermolecular β sheet between ATG3 residues 95 to 110 and β2 of LC3 in our structural model is consistent with the presence of a noncanonical LIR motif in the flexible region of ATG3, which was recently shown to be required for LC3 lipidation in cellulo (*37*). Combined with crystallographic structures (*21*, *38*) of ATG12-ATG5 in quaternary complex with a bound fragment of ATG3 and the N-terminal ATG5-binding domain of ATG16L1, we present an atomistic model of the full LC3 lipidation machinery consisting of the E3-like ATG12–ATG5-ATG16L1 complex bound to the E2-substrate conjugate, ATG3-LC3 (Fig. 1A).

To determine the configuration of the ATG12–ATG5-ATG16L1 complex recruited to phagophore membranes by the PI(3)P effector WIPI2, we first performed atomistic molecular dynamics (MD) simulations of a WIPI2-ATG16L1 cocrystal structure (*18*) in which WIPI2 is bound to the W2IR of ATG16L1 (residues 207 to 230). Initially placed at a minimum distance of ~2 nm above PI(3)P-containing membranes mimicking the endoplasmic reticulum (ER) lipid composition (*39*), WIPI2 established spontaneous membrane contacts in an expected orientation, with the two putative phosphoinositide binding sites (*40*–*42*) in its β-propeller blades 5 and 6 interacting with PI(3)P (Fig. 1A) and the N-terminal side of the bound ATG16L1 segment oriented away from the membrane. Lipid interactions were formed nearly exclusively in blades 5 and 6, around the conserved FRRG motif and in the 6CD loop (fig. S2). In extended MD simulations, we have previously demonstrated the ability of the 6CD region to form a membrane-inserting amphipathic helix that shows moderate curvature sensitivity (*25*). By aligning our structural model of ATG12–ATG5-ATG16L1 described above to the membrane-associated WIPI2-ATG16L1 configuration established during simulations, a first model of the membrane-recruited LC3 lipidation machinery was thus obtained.

Upon atomistic MD simulations of ATG12–ATG5-ATG16L1 complexed with the ATG3-LC3 conjugate and anchored via WIPI2 to membranes approximating the phagophore lipid composition, all components maintained structural integrity across all five 1-μs simulation replicates (fig. S3). Flexing and tilting motions of the dimeric coiled-coil structure of ATG16L1 were accompanied by considerable flexibility exhibited in the interdomain loop regions of ATG16L1 and ATG3 (Fig. 1B), allowing the ATG3-LC3 conjugate to explore favorable binding configurations near the membrane (movie S1). However, membrane binding was observed only in the case where ATG3-LC3 was already at the membrane surface upon initiation of the simulation. This finding indicates that the upward tilt of the WIPI2-attached coiled coil tends to keep the ATG3-LC3 conjugate above the membrane, even if direct interactions of ATG3-LC3 are possible in principle.

To reconcile the prevailing model of membrane recruitment of ATG12–ATG5-ATG16L1 by WIPI2 with the requirement for the membrane-interacting ATG16L1 N-terminal helix α2 (*26*), we hypothesized that upon initial recruitment through WIPI2, direct membrane binding by helix α2 constitutes a crucial second step in delivering ATG3-LC3 nearer to the target membrane. We focus here on the cis configuration in which the entire LC3 lipidation machinery becomes associated with the same patch of membrane (*43*). Our molecular model does not rule out the alternative possibility whereby ATG12–ATG5-ATG16L1 anchored at omegasomal membranes would bridge an intermembrane distance to facilitate LC3 conjugation to the nascent phagophore in trans (*22*). However, it seems difficult to reconcile the trans model with occupancy of the second WIPI2 site (*27*).

### Docking step 2: Helix α2 of ATG16L1 pulls ATG3-LC3 to membrane

**Fig. 2.**
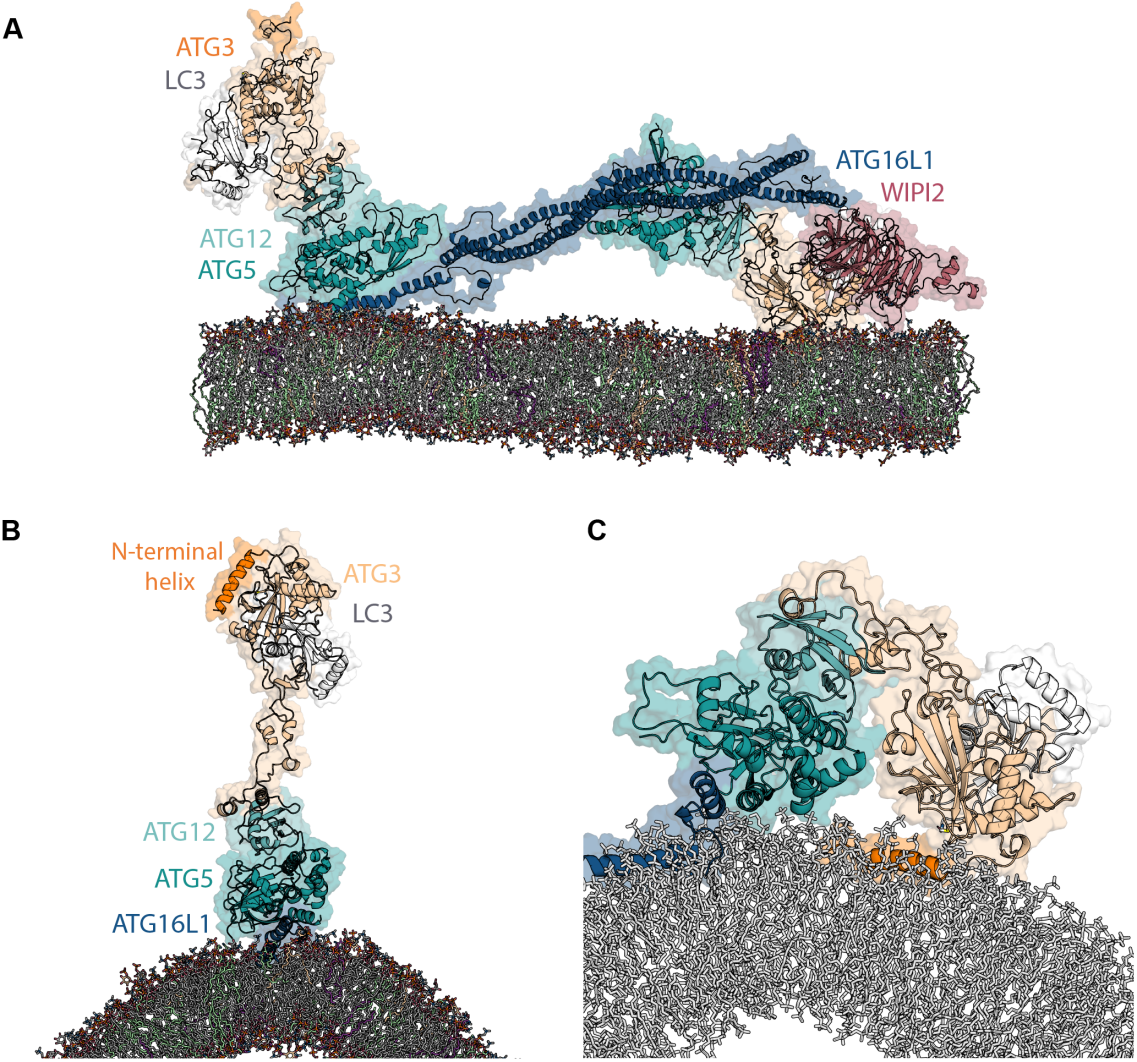
Membrane-bound ATG16L1 helix α2 brings ATG3-LC3 into membrane contact. (**A**) Snapshot of the WIPI2-recruited ATG12–ATG5-ATG16L1 complex with the N terminus of ATG16L1 also engaged in membrane interaction in an atomistic MD simulation. The ATG16L1 helix α2 had been steered gently to the membrane surface at a speed of 0.5 nm ns^−1^, by application of harmonic restraints on the center-of-mass distance between the helix and the membrane. The complex was subsequently relaxed in extended (1 μs) simulations. (**B**) Initial configuration of simulations representing a further stage of ATG3-LC3 delivery by ATG12–ATG5-ATG16L1, following membrane recruitment via WIPI2. The hydrophobic face of helix α2 is embedded into the membrane at this stage. (**C**) Snapshot of ATG3-LC3 delivered to the membrane while bound to ATG12–ATG5-ATG16L1, with the ATG3 N-terminal helix (orange) spontaneously inserted between membrane lipids. Taken at *t* = 450 ns from a 1-μs simulation replicate.

As a possible second step in membrane targeting, we focused on helix α2 of the ATG16L1 N-terminal domain, which has been shown to bind membranes (*26*) with a preference for positive membrane curvature (*25*). For an ATG12–ATG5-ATG16L1 complex attached to the membrane via WIPI2, this mode of membrane interaction is made possible by the flexibility of the ~30-residue interdomain loop between the N terminus of ATG16L1 and its coiled coil. We demonstrated this ability of ATG16L1 to engage with the membrane simultaneously, at one end, through recruitment by WIPI2 and, at the other end, via helix α2 by gently pulling the ATG16L1 helix α2 toward the membrane in steered MD simulations and then relaxing the complex in extended MD simulations (Fig. 2A).

Building upon our previous MD simulations of ATG12–ATG5-ATG16L1 binding to curved membranes (*25*), with ATG16L1 interacting either at the membrane surface or with an embedded hydrophobic face of the amphipathic helix α2, we obtained models of membrane-bound ATG12–ATG5-ATG16L1 loaded with ATG3-LC3 (Fig. 2B). In atomistic MD simulations, the flexible ATG3 loop then allowed ATG3-LC3 to reach the membrane spontaneously while maintaining its interactions with the α2-anchored ATG12–ATG5-ATG16L1 complex (movie S2). In this configuration, we found the N-terminal helix of the ATG3 conjugase to embed into the membrane (Fig. 2C and movie S2) without any biasing force. Once inserted, the ATG3 amphipathic helix remained embedded in the membrane through the course of the simulation, establishing stable membrane contacts. This finding is consistent with the previously reported role of the ATG3 N terminus as an essential membrane-targeting element (*44*). Geometrically, the two ATG3-LC3 conjugates flexibly connected to the ATG12–ATG5-ATG16L1 complex can simultaneously engage with the membrane for parallel lipidation reactions.

### Docking step 3: Catalytic domain of ATG3 forms stable membrane interaction interface upon membrane insertion of ATG3 N-terminal helix

**Fig. 3.**
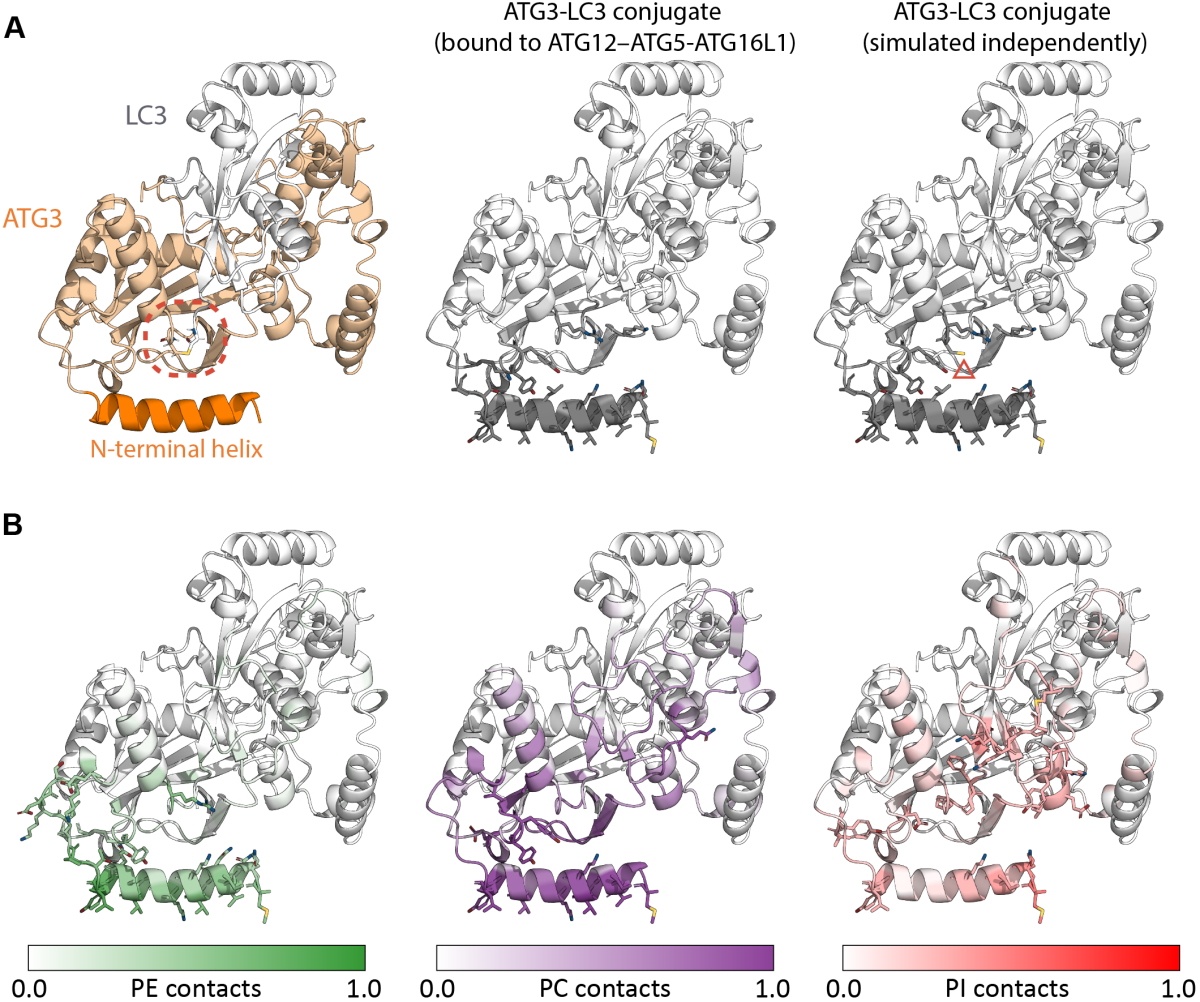
Membrane lipid contacts formed by the ATG3-LC3 conjugate. (**A**) Ribbon representation of the ATG3-LC3 conjugate structure before simulation, with the catalytic site indicated with a red dashed circle around the thioester bond (left). Coloring ATG3-LC3 residues by their mean frequency of membrane contacts (middle/right; white to gray at increasing contact frequency), upon spontaneous insertion of the ATG3 N-terminal helix in atomistic MD simulations, reveals a consistent membrane interaction interface while bound to ATG12–ATG5-ATG16L1 (final 500 ns of a 1-μs trajectory) and in the absence thereof (final 1 μs of each of eight 2-μs replicates). Top-ranked residues are highlighted as sticks: the only qualitatively discernible difference between membrane interaction data from the two independent simulation systems is indicated with a red triangle. For clarity of comparison, lipid interaction data are projected onto the same view of the initial model before simulation. (**B**) Proportion of membrane contacts formed by ATG3-LC3 residues with different types of lipids present in the membrane, illustrated with PE, phosphatidylcholine (PC), and PI. Membrane interaction data are averaged across the final 1 μs of eight 2-μs replicates and normalized for each lipid type such that a value of 1.0 is assigned to the residue(s) showing highest specificity for that lipid.

For a decisive third and final targeting step, we explored how the catalytic domain established membrane contact. The ATG3 conjugase has been reported to show basal activity in vitro for catalyzing LC3 conjugation in the absence of ATG12–ATG5-ATG16L1 (*45*). Having observed stable membrane association of the ATG3-LC3 conjugate held near phagophore-mimetic membranes by ATG12–ATG5-ATG16L1, we sought to further collect lipid contact data on ATG3-LC3 by initiating a set of longer (2-μs) replicates of smaller simulation systems containing the isolated conjugate placed directly above membranes. In 8 of the 20 trajectories thus obtained, spontaneous membrane insertion of the ATG3 N-terminal helix occurred within the first 1 μs. Comparison of ATG3-LC3 lipid contacts (after insertion of the ATG3 helix) reveals a consistent membrane interaction interface in the presence or absence of ATG12–ATG5-ATG16L1 (Fig. 3A and fig. S4). Membrane insertion of the N-terminal helix is also accompanied by its increased ordering relative to the enzyme body (fig. S5), consistent with a hypothesized role in controlling the structural dynamics of the complex (*46*). We found that the ATG3 protein dominated the interactions of the conjugate with PE-containing membranes.

### ATG3-LC3 presents active site toward the membrane in configuration conducive to lipidation reaction

With ATG3-LC3 at the membrane, we explored the structural foundation of the actual lipidation reaction. The folded core of ATG3 comprises a six-stranded β sheet (strands β1 to β6) surrounded by α helices (*47*). Among regions of ATG3-LC3 that formed frequent membrane interactions in our simulations were short sequences of residues within intersecondary structure loops of the ATG3 core, namely, (i) catalytic domain residues 208 to 211 and 242 to 243 of the β3/β4 and β4/β5 loops, respectively; (ii) residues 262 to 265 encompassing the thioester-forming Cys^264^ between β6 and the succeeding α helix; and to a lesser extent (iii) residues 61 to 64 within the β1/β2 loop. The catalytic site, which contains Cys^264^ of ATG3 covalently bonded to the C terminus of LC3, was situated centrally on the membrane interaction interface identified above and exposed toward membrane lipids (Fig. 3A). Furthermore, the ATG3-LC3 conjugate formed distinct interactions with different types of lipids present in the membrane, with PE localizing particularly near the catalytic center (Fig. 3B). Our data thus suggest a preferred orientation of ATG3-LC3 on the membrane that is compatible with catalyzing LC3 conjugation to the phagophore.

### Mutations at ATG3 membrane interaction face impair LC3 lipidation in vitro and in cellulo

**Fig. 4.**
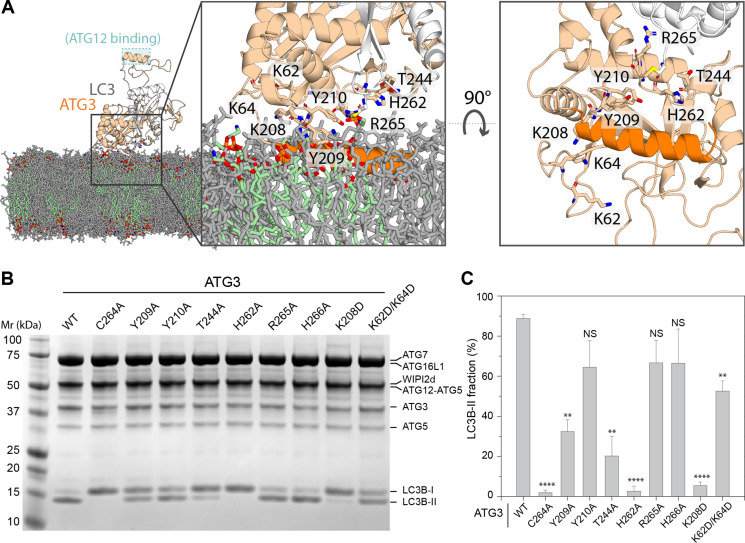
Mutational analysis of ATG3 membrane interaction face. (**A**) Snapshot of ATG3-LC3 upon spontaneous membrane association, taken at *t* = 1 μs of a 2-μs simulation replicate. Zoomed-in views from the side (left) and bottom (right; with lipids omitted for clarity) are shown. Phosphatidylethanolamine lipids are highlighted in green. (**B**) ATG3 in vitro LC3 lipidation. Do-SUVs [70% PC:20% PE:5% PS:5% PI(3)P] were incubated with ATG3 wild type or mutant, ATG7, E3, WIPI2d, and LC3B. After 20 min, samples were loaded onto a 4 to 15% SDS-PAGE gel and stained with Coomassie blue. (**C**) Quantification of in vitro LC3 lipidation results, plotting the LC3B-II percentage in the total band intensities of LC3B-I and LC3B-II. *P* values were calculated using Student’s *t* test: not significant (NS), *P* ≥ 0.05; **, 0.001 < *P* < 0.01; ****P < 0.0001.

The MD simulations identified a surface of ATG3 that was consistently in contact with the membrane in the context of the larger ATG12–ATG5-ATG16L1-ATG3–LC3B-WIPI2 complex. Residues in this patch include Lys^62^, Lys^64^, Lys^208^, Tyr^209^, Tyr^210^, Thr^244^, His^262^, Cys^264^, Arg^265^, and His^266^ (Fig. 4A). The presence of Cys^264^ was expected, given this residue’s known role as the LC3 donor in the reaction (*6*, *21*, *48*). We assayed LC3B conjugation activity in a small unilamellar vesicle (SUV) system similar to that originally used to demonstrate Atg8 conjugation activity of the Atg12–Atg5-Atg16-Atg3 complex (*48*). Here, purified human proteins were used (1.0 μM), WIPI2 (0.5 μM) was included in the protein mixture, and 10% PI(3)P was included in the SUVs (*18*). Activity was monitored by the conversion of LC3B-I to LC3B-II. As expected, essentially complete conversion was seen for wild type, while the mutation C264A of the catalytic cysteine as a negative control completely eliminated activity (Fig. 4, B and C). The mutation H262A also completely abolished activity, suggesting a direct role in catalysis beyond its membrane interactions alone. This is discussed further below. Activity was nearly abolished in K208D and sharply reduced in T244A, with small but significant reductions seen in K62D/K64D and Y209A. Smaller apparent reductions were seen in Y210A, R265A, and H266A. The observation that most of these mutations had at least some effect on catalysis in the SUV system confirms the predicted membrane interaction surface identified by the MD simulations.

**Fig. 5.**
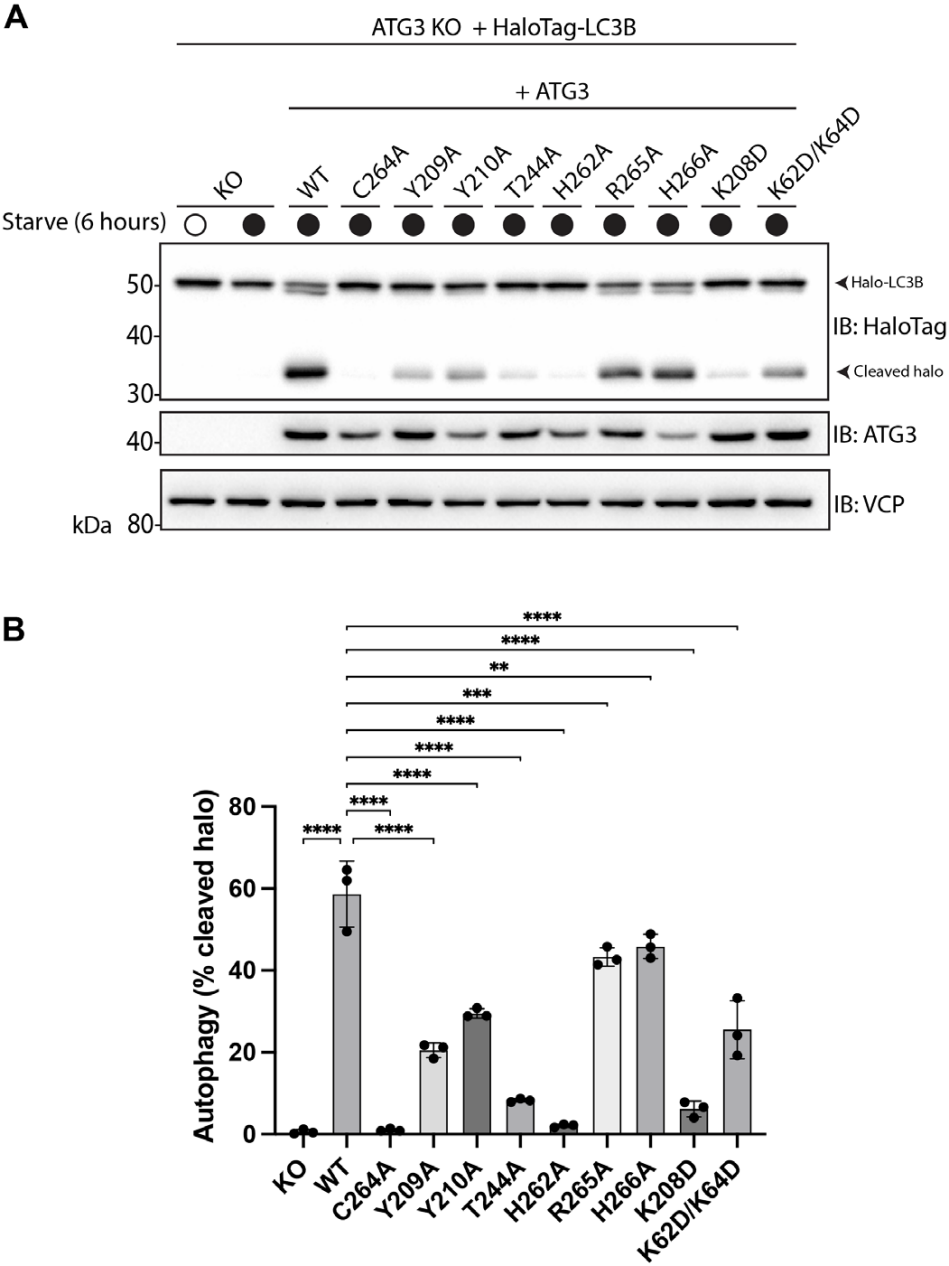
Mutations in ATG3 membrane interaction face impair function in cells. (**A**) ATG3 KO stably expressing HaloTag-LC3B with and without untagged ATG3 wild type (WT) or mutants were starved in EBSS for 6 hours. Cells were pulse labeled with 50 nM TMR-conjugated Halo ligand before starvation. Cell lysates analyzed by immunoblotting, showing one representative subset of data from triplicate experiments (fig. S6). (**B**) Autophagy levels represented by percentage cleaved Halo were obtained by calculating band intensities of free Halo (cleaved) compared to total Halo (uncleaved plus cleaved). Significance was calculated by comparing KO and mutants to WT. *P* values were calculated via one-way ANOVA: **P < 0.01, ***P < 0.001, and ****P < 0.0001. Data shown are mean ± SD from three independent experiments.

To determine whether the predicted membrane function had the same function in living cells as in the reconstituted system, we generated an ATG3 knockout (KO) HeLa cell line. ATG3 KO was verified by Western blotting (Fig. 5A). Starvation-induced autophagic flux was monitored with the HaloTag-LC3B system based on the appearance of a free HaloTag band (*17*). As expected, expression of the wild-type construct rescued autophagic flux in the KO cells, while no flux was observed in the C264A rescue (Figs 5, A and B, and fig. S6). The mutational effects on autophagic flux in the ATG3 KO cells mirror the pattern seen in the SUV assays. Y210A, R265A, and H266A, which have small (not statistically significant) reductions in activity in SUVs (Fig. 4, B and C), manifest modest reductions in flux in cells (Fig. 5, A and B). H262A and K208D show a nearly complete loss of activity in both the SUV (Fig. 4, B and C) and flux assays (Fig. 5, A and B). The effects of Y209A, T244A, and K62D/K64D are intermediate in both settings [Figs. 4 (B and C) and 5 (A and B)]. The rescue of conversion of LC3B and GABARAPL1 was also monitored in cells (fig. S6). Mutations that show a complete loss of activity in SUVs and the flux assay were also negative for LC3B and GABARAPL1 conversion. Mutations with intermediate defects in the SUV and flux assays showed smaller defects in the ATG8 protein conversion, which is attributed to differences in the stringency of the assays. The main conclusion from the ATG3 KO experiments is that the membrane interaction surface identified in the MD simulations accurately predicted loss of function in the biochemical and cellular assays.

### Conserved His^262^ of HPC motif facilitates ATG3-catalyzed LC3 lipidation

Previous studies have shown that the transfer of LC3 from ATG3 to lipid substrates is sensitive to pH and takes place more efficiently under slightly basic conditions in vitro, most likely through an effect on the ATG3 conjugase activity (*49*, *50*). While the protonation state of the target PE amine group is expected to show little variation within the pH range of interest, we note the presence of two histidine residues, His^262^ and His^266^, in close proximity to the catalytic Cys^264^. Both histidines are fully conserved across ATG3 homologs and, with their characteristic p*K*_a_ just below physiological pH, serve as possible acidity sensors for the ATG3-catalyzed reaction.

**Fig. 6.**
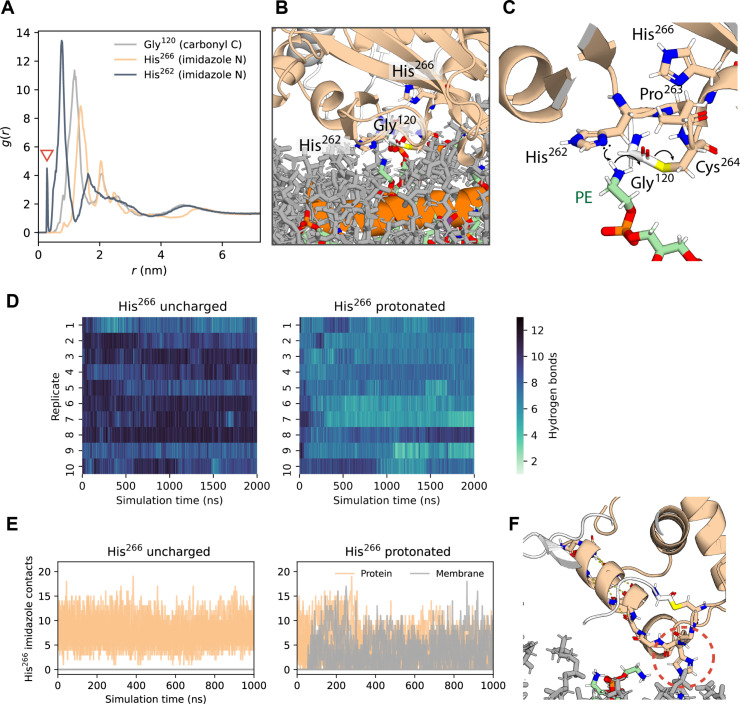
Two conserved histidine residues around ATG3 active site assume distinct roles. (**A**) Radial distribution function *g*(*r*) of the amine nitrogen atoms of PE lipids as a function of their distance *r* from select protein atoms (arrow: direct contact). (**B**) Snapshot of ATG3-LC3 interacting with membrane lipids during atomistic MD simulation. The zoomed-in view captures a PE lipid binding into the ATG3 active site, near the thioester bond under attack. The nearest PE amine proton is also within bonding distance (<0.2 nm) of the unprotonated nitrogen atom of the His^262^ imidazole ring. (**C**) Possible mechanism for initiation of LC3 lipidation reaction, whereby the ATG3 His^262^ imidazole ring would facilitate a nucleophilic attack on the Gly^120^ carbonyl of LC3. The backbone amide of Cys^264^ is in position to stabilize the developing negative charge on the Gly^120^ oxygen. Illustrated using the simulation snapshot of (B), showing only the “attacking” lipid for clarity. (**D**) Number of backbone hydrogen bonds in ATG3 helix 265 to 280 in simulations of ATG3-LC3 with ATG3 His^266^ in an uncharged or doubly protonated state. (**E**) Number of protein and membrane lipid contacts made by the His^266^ imidazole ring in the uncharged and the doubly protonated state, respectively, in simulations of alternative models of the ATG3-LC3 conjugate. (**F**) Snapshot of membrane-associated ATG3-LC3 in which the side chain imidazole of His^266^ (indicated with a red dashed circle) was doubly protonated with a charge of +*e*. At *t* = 1 μs of the 2-μs simulation replicate shown, destabilization of the local protein structure has brought the His^266^ side chain into membrane contact.

In atomistic MD simulations of the ATG3-LC3 conjugate with the His^262^ and His^266^ side chains both in their unprotonated state (which is predicted to be the dominant species at pH ≥ 7), His^266^ remained oriented toward the protein interior with a minimum distance of ~1 nm to the nearest lipid (Fig. 6A). By contrast, frequent lipid interactions formed by His^262^ are suggestive of a direct role in the LC3 conjugation reaction. Whereas the nucleophile of the reaction, the PE amine group, did spontaneously approach the backbone carbonyl carbon of Gly^120^ (LC3) to be attacked (reaching a minimum distance of ~0.4 nm), such interactions were infrequent. Meanwhile, the unprotonated nitrogen of the His^262^ imidazole was observed to interact with the positively charged primary amine of PE headgroups within bonding distance (<0.2 nm) to the amine proton (Fig. 6A). Furthermore, our simulations capture a configuration in which the His^262^:PE interaction coincided with that between PE and Gly^120^ (Fig. 6B).

His^262^ and Cys^264^ of human ATG3 form part of the HPC motif that is conserved across orthologs of ATG3 as well as ATG10, an E2-like autophagic enzyme that catalyzes ATG12 conjugation to ATG5 (*6*). Combined with previous (*35*, *51*) and present evidence of a critical role of His^262^ for ATG3 conjugase activity, our simulation results are suggestive of a plausible reaction mechanism in which the His^262^ imidazole ring would deprotonate the PE amine group for nucleophilic attack on the Gly^120^ carbonyl of LC3 (Fig. 6C). As part of such a proposed mechanism, the unique backbone conformational restraints conferred by the cyclic side chain of Pro^263^ in the HPC motif would be crucial for orienting His^262^ and Cys^264^ side chains in relative positions conducive to catalysis, explaining their full conservation (fig. S7). The protein backbone conformation conferred by Pro^263^ also holds the backbone amide of Cys^264^ within bonding distance of the carbonyl oxygen of Gly^120^ (Fig. 6C), which would stabilize the oxygen anion intermediate formed during the reaction. Energetically favorable breakage of the thioester bond will then yield the LC3-PE conjugate, a stable amide product. Alternatively, ATG3 has been reported to catalyze conjugation of ATG8 family proteins to phosphatidylserine (PS) lipids in the noncanonical pathway of autophagy (*52*). In accordance with this, our simulations of the ATG3-LC3 conjugate also capture an analogous membrane-interacting configuration likely poised for reaction with a PS molecule (fig. S8).

His^266^, the second of the two conserved histidine residues described above, has been implicated in the pH-dependent conjugase activity of ATG3 in a recent study (*35*). To assess the effect of altering the protonation state of His^266^, we performed additional MD simulations of the ATG3-LC3 conjugate in which the His^266^ imidazole ring was doubly protonated. Notably, the extra proton destabilized the local protein structure (Fig. 6D). A reorientation brought the His^266^ side chain into direct membrane contact within the first hundreds of nanoseconds in 7 out of 10 simulation replicates (Fig. 6, E and F). These results are consistent with His^266^ fulfilling a pH-sensitive structural role, as previously proposed for its counterpart in ATG3 orthologs (His^236^ in the yeast protein and His^260^ in *Arabidopsis*) (*50*), and provide an explanation for the alternative conformations in this region between available crystal structures obtained at different pH values (*47*, *53*).

## DISCUSSION

Building upon an increasing collection of structural and biochemical data on the components and interactions that form the autophagic LC3 lipidation machinery, we set out to complete the molecular puzzle of how the E3-like ATG12–ATG5-ATG16L1 complex and the E2-like conjugase ATG3 deliver LC3 to phagophore membranes. Results from atomistic MD simulations point toward a multistage mechanism progressively localizing the ATG3-LC3 conjugate nearer to the target membrane and orienting the reactive center of LC3 conjugation toward lipid substrates. This process requires the sequential action of three previously identified membrane sensors within the assembly: (i) WIPI2 as the PI(3)P effector protein that drives membrane recruitment of ATG12–ATG5-ATG16L1 (*22*, *54*), (ii) the curvature-sensitive ATG16L1 helix α2 within the (ATG12–)ATG5-binding domain (*25*, *26*), and (iii) the N-terminal amphipathic helix and membrane docking face of ATG3 (*44*).

As an emerging theme in cellular processes, with analogies to the multistep process of docking in vesicle fusion (*55*), the stepwise mechanism for the membrane targeting of LC3 provides additional layers of regulatory potential to the autophagic pathway. On the protein side, phosphorylation and other posttranslational modifications will affect the stability, accessibility, and affinity of the distinct interaction elements. On the membrane side, variations in lipid composition and phosphatidylinositol (PI) phosphorylation will modulate membrane recruitment. The phagophore lipid composition in particular modulates the recruitment of WIPI2 as anchor for ATG16L1 in docking step 1 as well as the membrane insertion of the ATG16L1 α2 helix and the ATG3 N-terminal helix in steps 2 and 3, respectively. Growing evidence points to a second WIPI2-interacting site within the ATG16L1 coiled-coil domain (*27*), which would facilitate step 2 of our model in a PI(3)P-dependent manner. Occupancy of the second site for WIPI2 has been proposed to facilitate LC3 lipidation following the initial membrane recruitment of ATG12–ATG5-ATG16L1, in line with earlier observations of allosteric activation of the complex by WIPI2 (*43*). Consistent with our structural model, a second WIPI2 molecule bound to the coiled-coil region would pull the N-terminal side of ATG16L1 closer to the membrane surface, with the WIPI2 FRRG motif oriented toward the membrane (fig. S9), thereby facilitating the membrane insertion of the ATG16L1 α2 helix in step 2 of our docking model. A three-dimensional model of the complex of ATG12–ATG5-ATG16L1 loaded with ATG3-LC3 anchored to the bilayer by a second WIPI2 (fig. S9) favors lipidation in cis.

Through multi-microsecond all-atom MD simulations, collecting a total of >50-μs membrane docking trajectories, we have examined the lipid-interacting regions of the complete ATG3-LC3 conjugate for molecular determinants of the LC3 conjugation mechanism. The spontaneous membrane insertion of the ATG3 N-terminal helix in our simulations is consistent with a role of this region in positioning the protein onto the membrane for subsequent enzymatic activity (*15*, *46*, *56*). Additional membrane-interacting residues concentrate around the Cys^264^ residue holding LC3. Mutations of these residues impact lipidation both in vitro and in vivo, confirming the catalytic relevance of the observed membrane interactions.

Simulations and experiments identify distinct roles for two fully conserved histidine residues in the vicinity of the catalytic cysteine of ATG3. We found neutral His^266^ to stabilize a catalytically competent structure of the active site, consistent with retention of full lipidation activity by the H266A mutant. In contrast, protonation of His^266^ disrupted the active site in our MD simulations, consistent with a role of His^266^ as pH sensor (*35*). While uncharged His^266^ serves to stabilize the catalytic loop conformation, our data point to active participation of His^262^ in the initiation of the LC3 lipidation reaction. In particular, we found the unprotonated His^262^ imidazole nitrogen to be positioned as proton acceptor from PE. Consistent with a possible catalytic role, the H262A mutation abolished function. His^262^ is the starting residue in the highly conserved HPC motif (*6*) of ATG3, which is shared with the ATG10 conjugase family. However, in ATG10, the counterpart of ATG3 His^266^ is a threonine, which may reflect the distinct substrate specificity of the two enzymes (fig. S7).

The critical biological role of the ATG12–ATG5-ATG16L1 complex in mammalian autophagy (*19*), and before that, the role of the corresponding Atg12–Atg5-Atg16 complex in yeast (*16*), has long been appreciated. Yet, the precise role of this complex in LC3 lipidation has been challenging to define. The role of the extensive structural elements linking the N-terminal helix of ATG3 on the one hand, and the established WIPI2-dependent membrane docking site on the other, have proven difficult to characterize as the membrane-associated system is too large for nuclear magnetic resonance, yet too dynamic for x-ray crystallography or single-particle cryo–electron microscopy. Under the “computational microscope” of MD simulations, the role of the connecting elements in mediating a stepwise docking process has now been unveiled. As a core element in the molecular machinery of selective autophagy, this far more detailed insight into the membrane docking steps of LC3 will undoubtedly facilitate the therapeutic targeting of autophagy in Parkinson’s disease and other neurodegenerative diseases.

## MATERIALS AND METHODS

### Structural models of protein complexes

Atomistic models of the ATG3-ATG12–ATG5-ATG16L1 and WIPI2d-ATG16L1 complexes were based on crystal structures with Protein Data Bank (PDB) IDs 4NAW (*21*) and 7MU2 (*18*), respectively. The ATG16L1 N-terminal domain in the former complex was replaced by a more complete structure [PDB ID: 4TQ0 (*57*)]. As introduced previously (*25*), an alternative conformation of the same ATG16L1 region was generated in PyMOL 2.3 (RRID:SCR_000305, https://pymol.org/) (*58*) by rotation of helix α2 relative to helix α1 at the Gln^30^/Ala^31^ hinge. A model for the dimeric central ATG16L1 domain was completed through (i) homology modeling of residues 141 to 225 using SWISS-MODEL (RRID:SCR_018123, https://swissmodel.expasy.org/) (*59*) based on crystal structures of the mouse protein [PDB IDs: 6ZAY (*32*) and 6SUR (*31*)] and (ii) parameter fitting for residues 78 to 193 with CCBuilder 2.0 (https://github.com/woolfson-group/ccbuilder2) (*29*) upon coiled-coil prediction (*28*) by NPS@ (https://npsa-prabi.ibcp.fr/) (*60*). Unstructured interdomain loops were added using the DEMO server (https://zhanggroup.org/DEMO/) (*61*) to yield an ATG16L1 dimer encompassing residues 1 to 247. AlphaFold v2.2 (https://github.com/google-deepmind/alphafold) (*34*) was used to model ATG3-LC3B in complex with the ATG7 homodimer. The Cys^264^ side chain of ATG3 was connected to the LC3B C terminus by a thioester bond, parameterized using CHARMM-GUI (https://charmm-gui.org/) (*62*, *63*). The ATG5 Lys^130^ side chain was similarly connected to the ATG12 C terminus, via an isopeptide bond. The ATG16L1 WD40 domain [dispensable for canonical autophagy (*64*)] was excluded from the model, as were the unstructured ATG12 residues 1 to 52 and WIPI2d residues 1 to 11 and 362 to 425. Exposed N- or C-terminal groups at the end(s) of each incomplete structure or truncated construct were neutralized. Protonation states of amino acid side chains were assigned according to p*K*_a_ prediction by PROPKA 3 (https://github.com/jensengroup/propka) (*65*). His^183^ and His^255^ at the putative PI(3)P binding sites of WIPI2d were protonated. Six models of the ATG3-LC3B conjugate were generated, with the imidazole of ATG3 His^262^ uncharged (protonated at the δ- or ϵ-nitrogen in alternative models) and that of His^266^ uncharged (protonated at δ- or ϵ-nitrogen in alternative models) or cationic (doubly protonated).

### MD simulations

MD simulations were performed with GROMACS 2020 (RRID:SCR_014565, http://gromacs.org/) (*66*) using the CHARMM36m force field (*67*). Following the same protocol as previously described (*25*), all membranes consisted of 60% dioleoylphosphatidylcholine (DOPC), 20% dioleoylphosphatidylethanolamine (DOPE), 5% dioleoylphosphatidylserine (DOPS), 10% 1-palmitoyl-2-oleoyl-*sn*-glycero-3-phosphoinositol (POPI), and 5% PI(3)P based on the ER lipid composition (*39*) and were prepared initially in a coarse-grained representation using the insane method (https://github.com/Tsjerk/Insane) (*68*). Curved membranes were constructed using LipidWrapper (https://github.com/durrantlab/lipidwrapper) (*69*) by fitting the amplitude of the membrane buckle as a sine function of its *x* coordinate. Each coarse-grained membrane system was solvated with 150 mM of aqueous NaCl, equilibrated for 200 ns and converted into an atomistic representation using the CG2AT2 (https://github.com/owenvickery/cg2at) (*70*) tool. Atomistic models of protein complexes were placed above membranes after CG2AT2 conversion, followed by resolvation and 10 ns of further equilibration. Simulation replicates were independently prepared and equilibrated. During equilibration, harmonic positional restraints with a force constant of 1000 kJ mol^−1^ were applied to nonhydrogen protein atoms or backbone beads. The *xy* dimensions of buckled membrane systems were fixed in simulations. System temperature and pressure were maintained at 310 K and 1 bar, respectively, using the velocity-rescaling thermostat (*71*) and a semi-isotropic Parrinello-Rahman barostat (*72*) during the production phase. The integration time step was 2 fs. Long-range electrostatic interactions were treated using the smooth particle mesh Ewald method (*73*, *74*) with a real-space cutoff of 1 nm, a Fourier spacing of 0.12 nm, and charge interpolation through fourth-order B splines. The LINCS linear constraint solver (LINCS) algorithm was used to constrain covalent bonds involving hydrogen atoms (*75*). Simulation trajectories (table S1) were analyzed through the MDAnalysis 2.0 library (https://mdanalysis.org/) (*76*, *77*) in Python 3.6 (RRID:SCR_008394, http://python.org/).

### Protein expression and purification

ATG3 constructs used for in vitro lipidation assays were expressed in *Escherichia coli* (BL21) DE3 star cells (Invitrogen, C601003). Cells were grown in LB media at 37°C until an OD_600_ (optical density at 600 nm) of 0.8 is reached. The culture was induced with 1 mM isopropyl-β-d-thiogalactopyranoside and grown overnight at 18°C. Cells were pelleted and resuspended in 50 mM Hepes (pH 7.5), 300 mM NaCl, 2 mM MgCl_2_, 10 mM imidazole, and 1 mM tris(2-carboxyethyl)phosphine (TCEP) supplemented with EDTA-free protease inhibitors (Roche). The cells were lysed via sonication, and lysate was clarified by centrifugation (17,000 rpm for 1 hour at 4°C). The supernatant was then applied to 1 ml of Ni–nitrilotriacetic acid resin. The resin was subsequently washed thoroughly with at least 100 column volumes (CV) of lysis buffer, and the protein was eluted with lysis buffer supplemented with 300 mM imidazole. The eluted proteins were concentrated and loaded onto a Superdex 200 column (10/300 GL; GE Healthcare) equilibrated with a buffer containing 25 mM Hepes (pH 7.5), 150 mM NaCl, and 1 mM TCEP. Peak fractions corresponding to the protein were collected, pooled, snap-frozen in liquid nitrogen, and stored at −80°C. Purification of ATG12–ATG5-ATG16L1 (dx.doi.org/10.17504/protocols.io.br6qm9dw), ATG7 (https://dx.doi.org/10.17504/protocols.io.bsennbde), and LC3 (https://dx.doi.org/10.17504/protocols.io.j8nlkw82dl5r/v1) used for liposome lipidation assays was performed as previously described (*43*). Purification of WIPI2d was performed as previously described (https://dx.doi.org/10.17504/protocols.io.buxqnxmw) (*18*).

### In vitro LC3 lipidation assays

A lipid mixture with a molar composition of 70% DOPC, 20% DOPE, 5% DOPI(3)P, and 5% DOPS (Avanti Polar Lipids) was dried under a nitrogen stream and put under vacuum overnight. Lipids were resuspended at 1 mg/ml in the assay buffer [25 mM Hepes (pH 7.5), 135 mM NaCl, 2.7 mM KCl, and 1 mM TCEP], freeze-thawed seven times, and extruded 17 times through a 100 nM filter (Whatman). Reactions were set up at room temperature in the assay buffer to a final concentration of 1 μM of the indicated ATG3 construct, 1 μM ATG7, 1 μM E3, 500 nM WIPI2d, 5 μM LC3B, 0.5 mM adenosine 5′-triphosphate, 1 mM MgCl_2_, and liposomes (0.5 mg/ml). Fifteen microliters of reaction was quenched at 20 min with 4× lithium dodecyl sulfate (LDS loading buffer, boiled at 60°C for 10 min, and then loaded onto SDS–polyacrylamide gel electrophoresis (SDS-PAGE) gels. Protein bands were visualized with Coomassie blue. Three biological replicates were performed. Protein band intensity of LC3B-I and LC3B-II was analyzed by ImageJ (RRID:SCR_003070, https://imagej.nih.gov/ij/). Quantification of LC3B-II formation was plotted as percentage of total LC3B among the measured values for each ATG3 protein in a bar graph. Averages and SDs were calculated. The *P* values were calculated using an unpaired two-tailed Student’s *t* test. *P* values were considered as follows: not significant (NS), *P* ≥ 0.05; *, 0.01 < *P* < 0.05; **, 0.001 < *P* < 0.01; ***, 0.0001 < *P* < 0.001; and ****P < 0.0001. The LC3 lipidation assay protocol is available at https://dx.doi.org/10.17504/protocols.io.e6nvwjxodlmk/v1.

### Cloning and generation of stably expressing ATG3 wild type and mutant HeLa cell lines

All HeLa cells (American Type Culture Collection, catalog no. CCL-2, RRID:CVCL_0030) used were cultured in Dulbecco’s modified Eagle’s medium (DMEM) supplemented with 10%(v/v) fetal bovine serum (Cell Sera), 10 mM Hepes, 1% (v/v) penicillin/streptomycin antibiotic solution (Sigma-Aldrich; P4333-100ML), 1× GlutaMAX (Gibco, 35050061), and 1× nonessential amino acids (Gibco, 11140050). All cells were stored under standard conditions in an appropriate vessel in a humidified incubator at 37°C and a CO_2_ level of 5%. Polymerase chain reaction products of ATG3 wild type and ATG3 mutants were subcloned into linearized pMX-IG backbone using NEBuilder HiFi DNA Assembly Master Mix (New England Biolabs, E2621L) containing an internal ribosomal entry site–yellow fluorescent protein element for untagged expression of ATG3 wild type and mutants. From this, the following ATG3 plasmids were generated: pMX-IG-ATG3 (RRID:Addgene 212021), pMX-IG-ATG3-C264A (RRID:Addgene 212023), pMX-IG-ATG3-Y209A (RRID:Addgene 212024), pMX-IG-ATG3-Y210A (RRID:Addgene 212025), pMX-IG-ATG3-T244A (RRID:Addgene 212026), pMX-IG-ATG3-H262A (RRID:Addgene 212027), pMX-IG-ATG3-R265A (RRID:Addgene 212028), pMX-IG-ATG3-H266A (RRID:Addgene 212029), pMX-IG-ATG3-K208D (RRID:Addgene 212030), and pMX-IG-ATG3-K62D/K64D (RRID:Addgene 212031). All plasmids were verified by DNA sequencing. Stable cell lines were generated using a retroviral system where pMRX-IP-HaloTag7-LC3 (RRID:Addgene 184899) and all pMX-IG ATG3 (RRID:Addgene 212021) constructs alongside retroviral packaging plasmids vesicular stomatitis virus glycoprotein (RRID:Addgene 8454) and Gag-pol (RRID:Addgene 14887) were transfected into human embryonic kidney 293T cells (RRID:CVCL_0063) using Lipofectamine LTX (Invitrogen, 15338030) for 15 hours. The next day, transfection medium was replaced with complete DMEM. After 24 hours, the retroviral supernatant was collected, filtered, and added to ATG3 KO HeLa cells for 24 to 48 hours alongside polybrene (8 μg/ml; Sigma-Aldrich, H9268). After transduction, cells were allowed to recover in full growth medium for 5 to 7 days before fluorescence sorting for positive cells via fluorescence-activated cell sorting. The protocol used for generating stable cell lines using the retroviral system is available at dx.doi.org/10.17504/protocols.io.81wgbyez1vpk/v1.

### HaloTag-LC3B starvation assay

Halo-LC3B Assay was performed as previously described (*78*). Cells were seeded at 400,000 cells per well in a six-well plate 1 day before. Cell were fed with 1 ml of complete DMEM for 1 hour followed by incubation in complete DMEM containing 50 nM tetramethylrhodamine (TMR)-conjugated Halo ligand (Promega, GA1120) for 20 min. Cells were then washed thrice with 1× PBS followed by incubation in Earle's Balanced Salt Solution (EBSS) buffer (Thermo Fisher Scientific, 2410043) to induce autophagy by starvation for 6 hours. Afterward, cells were washed with 1× PBS before harvesting with cell scrapers. Cells were pelleted and lysed in 1× LDS sample buffer (Invitrogen, NP0007) supplemented with 100 mM dithiothreitol (Sigma-Aldrich, 10708984001). Samples were heated at 99°C with shaking for 10 min, and protein concentration was measured using a nanodrop spectrophotometer (Thermo Fisher Scientific). Twenty micrograms of protein per sample was analyzed on 4 to 12% bis-tris gels (Invitrogen, WG1402A) according to the manufacturer’s instructions. Gels were electro-transferred to polyvinyl difluoride membranes (Immobilon) and immunoblotted using indicated antibodies. Quantification of percentage autophagy was calculated by measuring the amount of cleaved Halo against total Halo (cleaved plus uncleaved). Statistical analysis was performed via one-way analysis of variance (ANOVA) using the multiple comparisons function in GraphPrism 9 (RRID:SCR_002798, http://graphpad.com/). A complete protocol is available at dx.doi.org/10.17504/protocols.io.e6nvwdo9zlmk/v1.
