# Supplementary material for: Three-step docking by WIPI2, ATG16L1, and ATG3 delivers LC3 to the phagophore
Source: Sci Adv. 2024 Feb 7;10(6):eadj8027. doi: 10.1126/sciadv.adj8027 (PMC10851258; doi:10.1126/sciadv.adj8027)
Supplement: Supplementary file 1 — Figs. S1 to S9 Table S1 Legends for movies S1 and S2 [file sciadv.adj8027_sm.pdf]

Science Advances
AAAS

Supplementary Materials for

Three-step docking by WIPI2, ATG16L1, and ATG3 delivers LC3 to the phagophore

Shanlin Rao *et al.*

Corresponding author: Michael Lazarou, lazarou.m@wehi.edu.au; James H. Hurley, jimhurley@berkeley.edu; Gerhard Hummer, gerhard.hummer@biophys.mpg.de

Sci. Adv. **10**, eadj8027 (2024)
DOI: 10.1126/sciadv.adj8027

The PDF file includes:

Figs. S1 to S9
Table S1
Legends for movies S1 and S2

Other Supplementary Material for this manuscript includes the following:

Movies S1 and S2

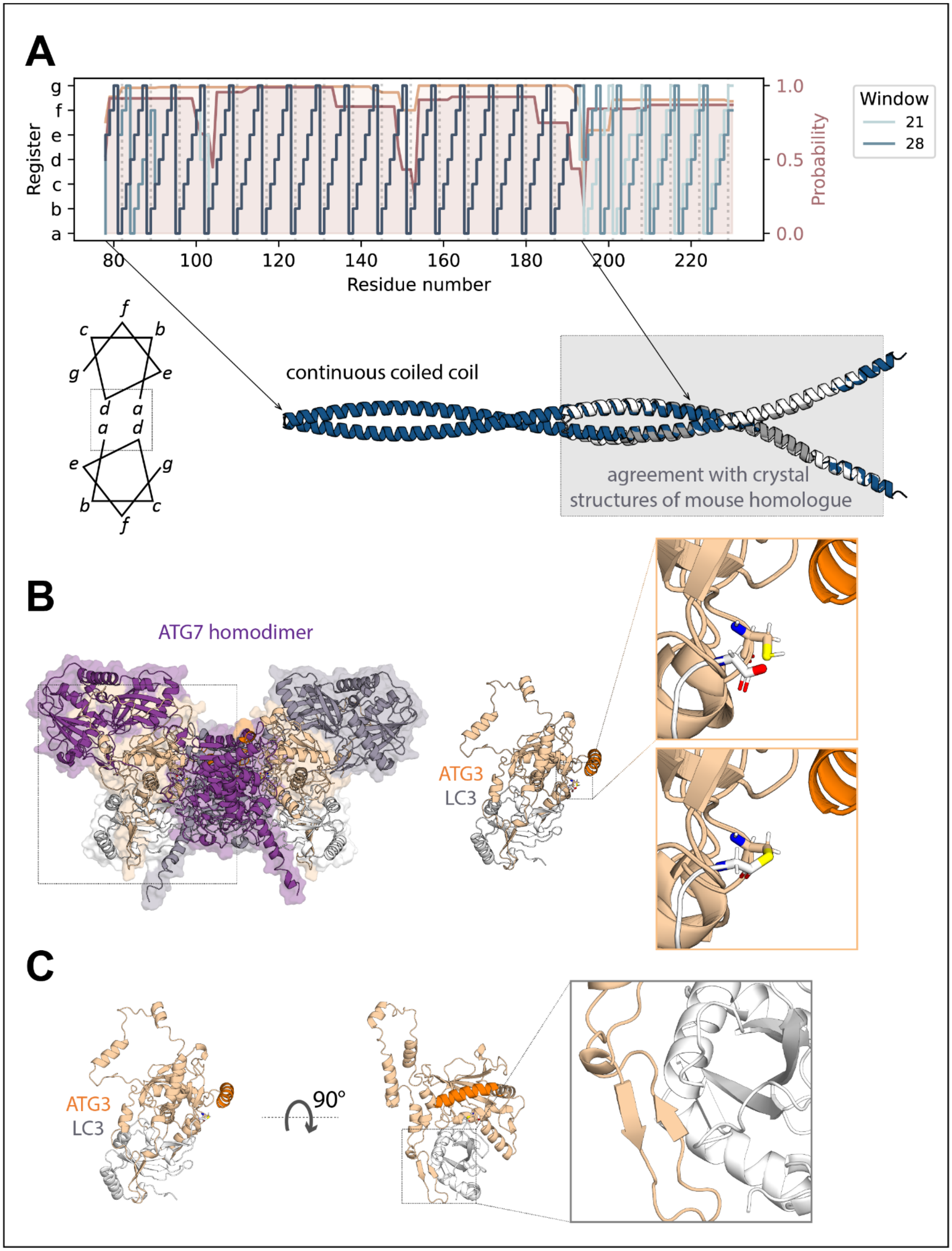
A
Register
g
f
e
d
c
b
a
1.0
0.5
0.0
Probability
Window
21
28
80
100
120
140
160
180
200
220
Residue number
continuous coiled coil
agreement with crystal structures of mouse homologue
B
ATG7 homodimer
ATG3
LC3
C
ATG3
LC3
90°

Fig. S1

Structural modeling of the human LC3 lipidation machinery components. (**A**) Coiled-coil prediction and modeling of the central homodimerization domain of ATG16L1. The C-terminal region of the predicted stretch of continuous coiled coils (residues 78-193) is in excellent agreement with homology models based on crystal structures of the mouse orthologue (PDB IDs: 6ZAY and 6SUR, shown in white and gray, respectively) in which an overlapping region has been resolved. (**B**) Structure of ATG7 homodimer in complex with two copies of ATG3-LC3, as predicted by AlphaFold. As part of the larger complex, the catalytic Cys264 of ATG3 is in close proximity to the C-terminal Gly120 of LC3. A rotameric modification of the Gly120 conformation allows a thioester bond to be modeled, yielding the ATG3-LC3 conjugate. (**C**) Predicted formation of intermolecular β-sheet between ATG3 residues 95-110 and β2 of LC3.

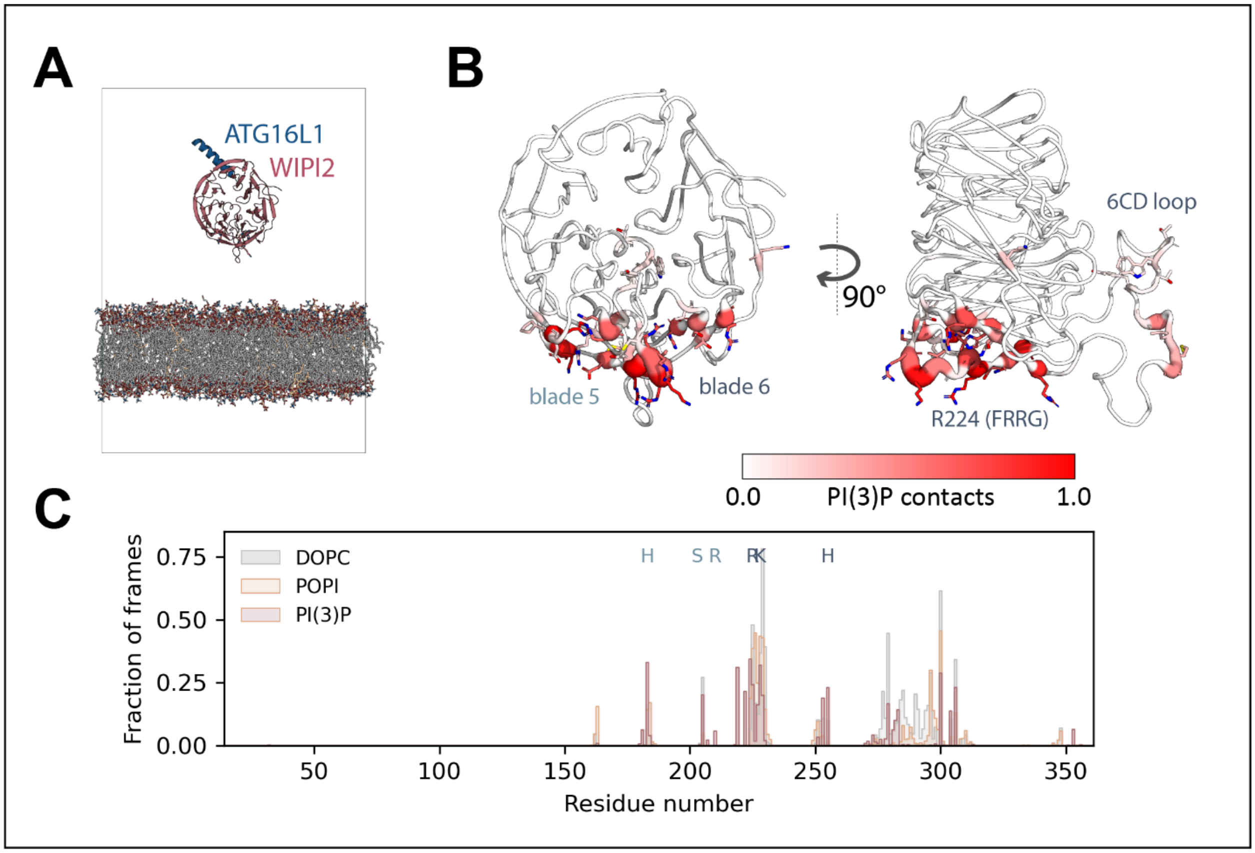

Fig. S2

Spontaneous lipid interactions of WIPI2. (**A**) Initial configuration of WIPI2 in complex with ATG16L1 residues 207-230 above PI(3)P-containing membranes, prior to atomistic molecular dynamics simulations. Water and ions solvating the system are not shown, for clarity. (**B**) Putty representation of the initial WIPI2 configuration, colored by the mean frequency of PI(3)P contacts formed by WIPI2 residues over five 300 ns simulations. Positions of the β-propeller blades 5 and 6 are indicated. (**C**) Mean frequency of PI(3)P contacts formed by WIPI2 residues in comparison with contacts with POPI and DOPC lipids. Blade 5 and blade 6 residues constituting the two putative phosphoinositide binding sites are indicated.

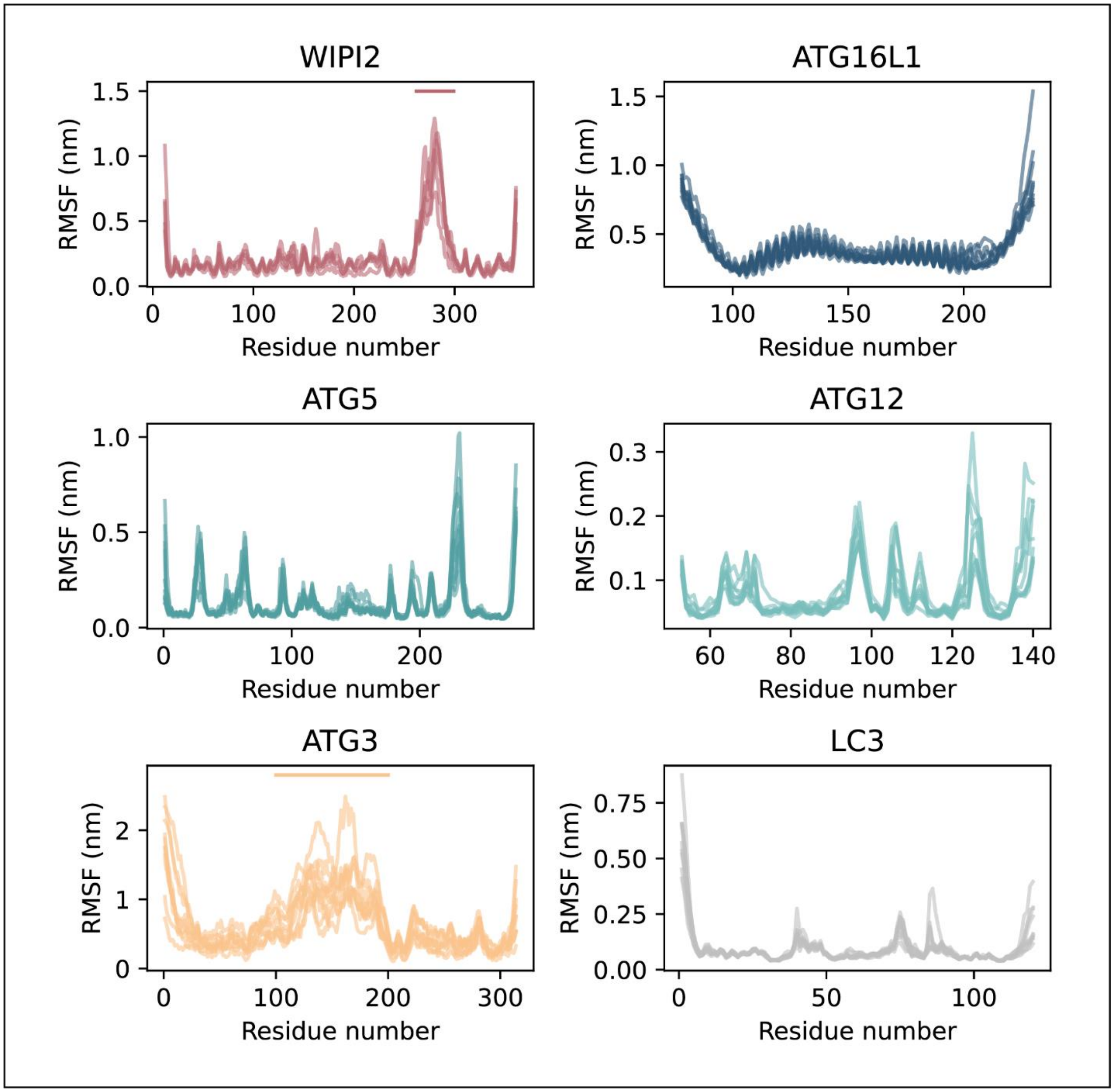

Fig. S3

Root-mean-square fluctuation (RMSF) assessment of protein stability. Cα RMSFs of the membrane-associated ATG12–ATG5-ATG16L1-WIPI2 complex loaded with ATG3-LC3 are plotted for the respective constituent subunits during five 1 μs simulation replicates. Extended loop regions along the WIPI2 and ATG3 sequence are each indicated with a horizontal line.

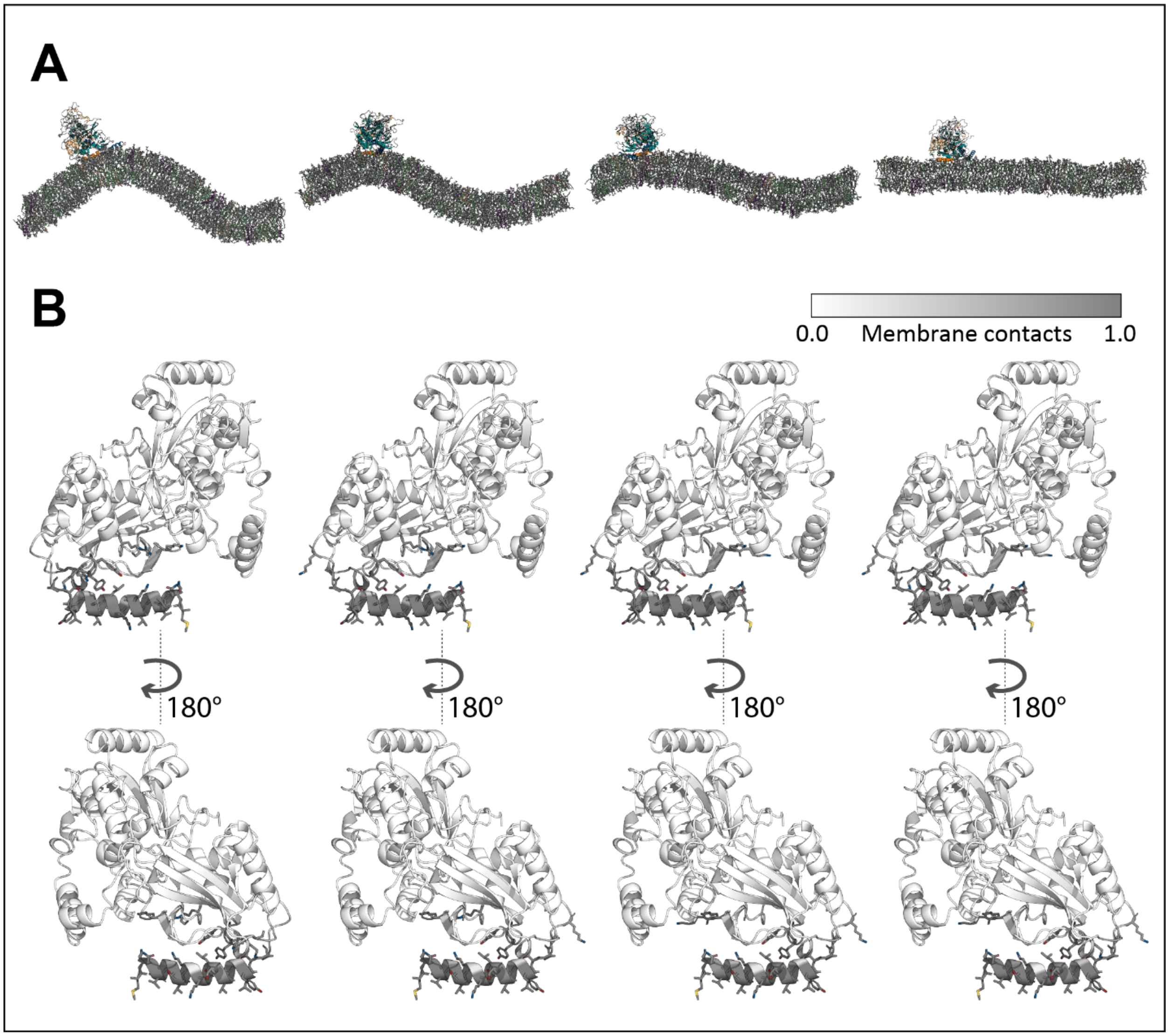

Fig. S4

Membrane lipid contacts formed by the ATG3-LC3 conjugate bound to ATG12–ATG5-ATG16L1 on membranes with different degrees of local curvature. (**A**) Snapshots taken at *t* = 1 μs of independent all-atom molecular dynamics simulations of ATG12–ATG5-ATG16L1-ATG3–LC3 on membranes ranging from highly curved to flat. Over three 1 μs simulation replicates, the mean local membrane curvature *H* sampled by the construct had mean values of ~0.065 nm^{-1}, ~0.05 nm^{-1}, ~0.03 nm^{-1} and < 0.01 nm^{-1} in the four systems, respectively. Water, ions, and a second copy of the protein complex bound to the opposite leaflet are not shown. (**B**) Ribbon representation of ATG3-LC3 colored by the mean frequency of membrane contacts formed by ATG3 or LC3 residues during the final 500 ns of 1 μs simulations on membranes with four different levels of curvature, corresponding to systems depicted in (**A**). For clarity of comparison, lipid interaction data are projected onto the same view of the initial model prior to simulation, revealing a near-identical interaction interface, irrespective of membrane curvature.

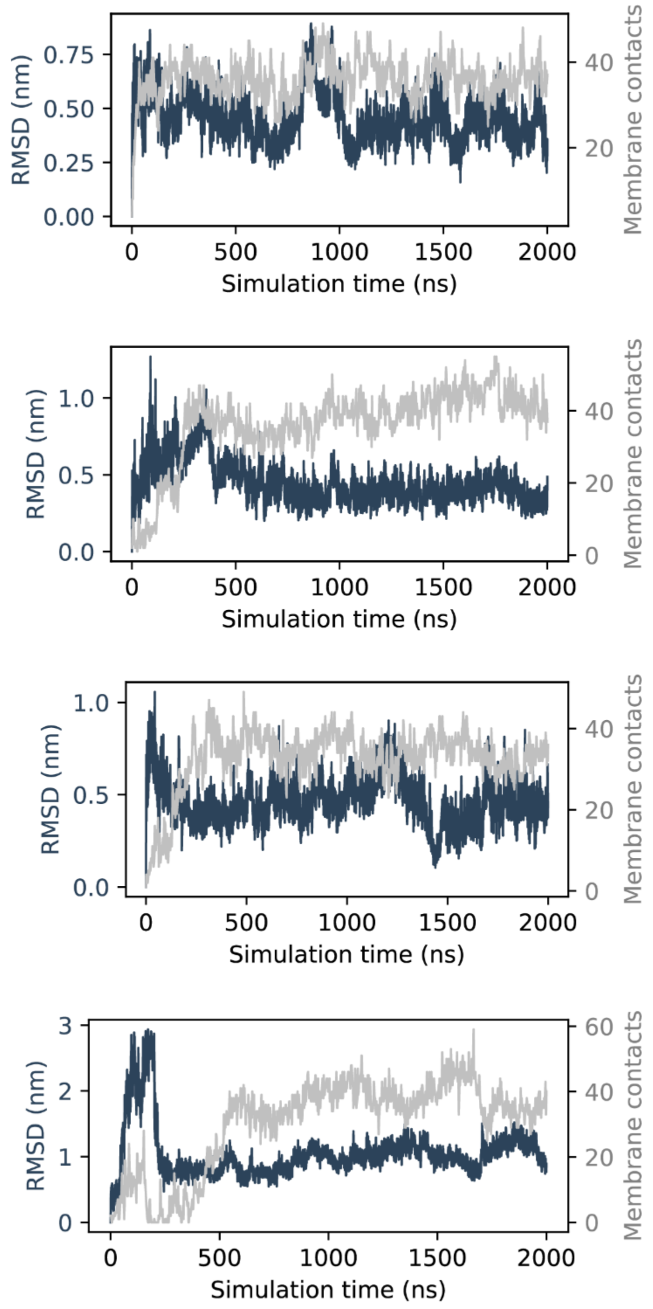

Fig. S5

Membrane insertion of the ATG3 N-terminal helix was accompanied by increased ordering in its positioning relative to the enzyme body. Cα root-mean-square deviations (RMSD, blue) of ATG3 residues 4-19, calculated upon least-squares fitting of the enzyme body to its initial conformation, are plotted alongside the number of ATG3 membrane contacts (gray) for four 2 μs simulation trajectories.

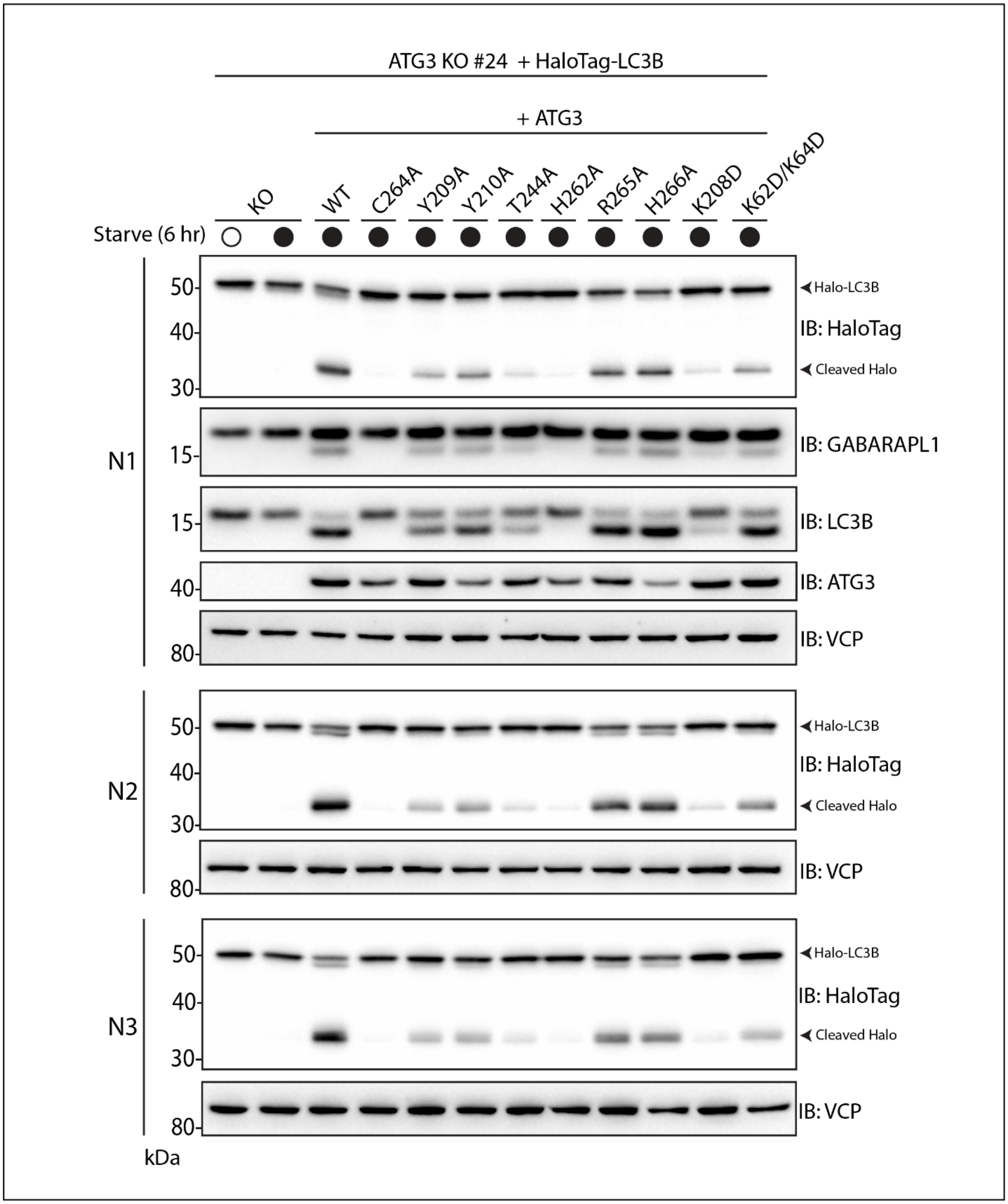

Fig. S6

Mutations in ATG3 membrane interaction face impair function in cells. ATG3 KO stably expressing HT-LC3B with and without untagged ATG3 wild-type (WT) or mutants were starved in EBSS for 6 hours. Cells were pulse labelled with 50 nM TMR-conjugated Halo ligand prior to starvation. Cell lysates were analysed by immunoblotting. All blots are grouped by repeat. Replicate N2 also shown in Fig. 5A.

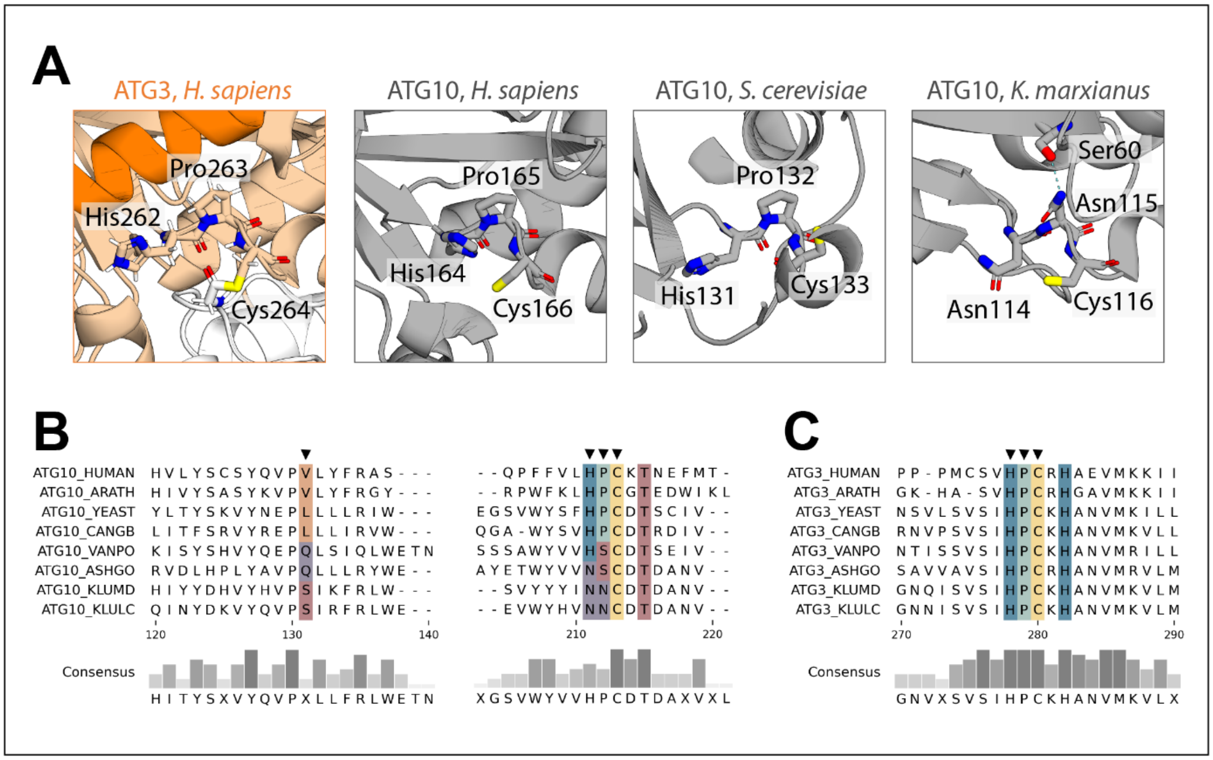

Fig. S7

Conservation of the HPC motif in the active site of ATG3 and ATG10 conjugase enzymes. (**A**) Structure of the human ATG3 His262, Pro263, and Cys264 motif and its counterparts in the predicted structure of human ATG10 (from AlphaFold) and in crystal structures of two fungal homologs (PDB IDs: 4EBR and 3VX7), respectively. In each case, the cysteine and histidine (or asparagine) of the motif are held in the same relative orientation by the peptide backbone, stabilized either by the unique cyclic side chain of the proline or, in the case of the *K. marxianus* protein, through additional hydrogen bonding. (**B**) Sequence alignment of ATG10 orthologs reveals that substitution of the HPC motif proline by a serine or the larger asparagine residue is invariably accompanied by a corresponding mutation (from a hydrophobic residue to a glutamine or the smaller serine, respectively) at a position further upstream. The resulting formation of a serine-glutamine or asparagine-serine hydrogen bond would stabilize the peptide backbone of the motif in place of a proline. (**C**) Sequence alignment of ATG3 orthologs from the same selection of organisms.

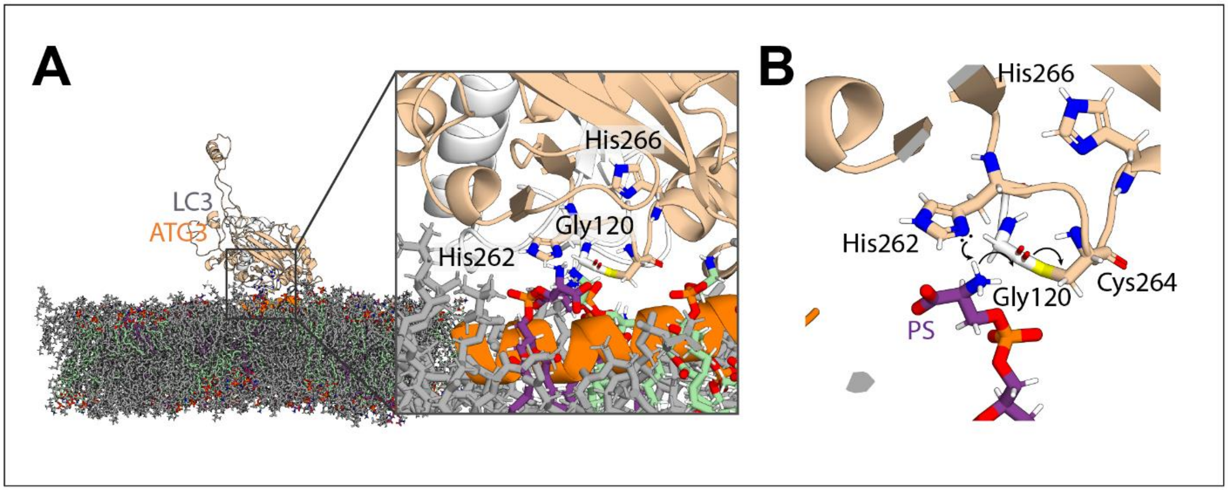

Fig. S8

Configuration of ATG3-LC3 conjugate poised for reaction with phosphatidylserine (PS) in atomistic molecular dynamics simulation. (**A**) Snapshot of ATG3-LC3 interacting with membrane lipids, capturing a PS lipid binding into the ATG3 active site, near the thioester bond under attack. The nearest PS amine proton also interacts within bonding distance (< 0.2 nm) of the unprotonated nitrogen atom of the His262 imidazole ring. (**B**) Possible mechanism for initiation of LC3 lipidation reaction, whereby the ATG3 His262 imidazole ring would facilitate nucleophilic attack on the Gly120 carbonyl of LC3. The backbone amide of Cys264 is in position to stabilize the developing negative charge on the Gly120 oxygen. Illustrated using the same simulation snapshot from (**A**), omitting all lipids but one for clarity.

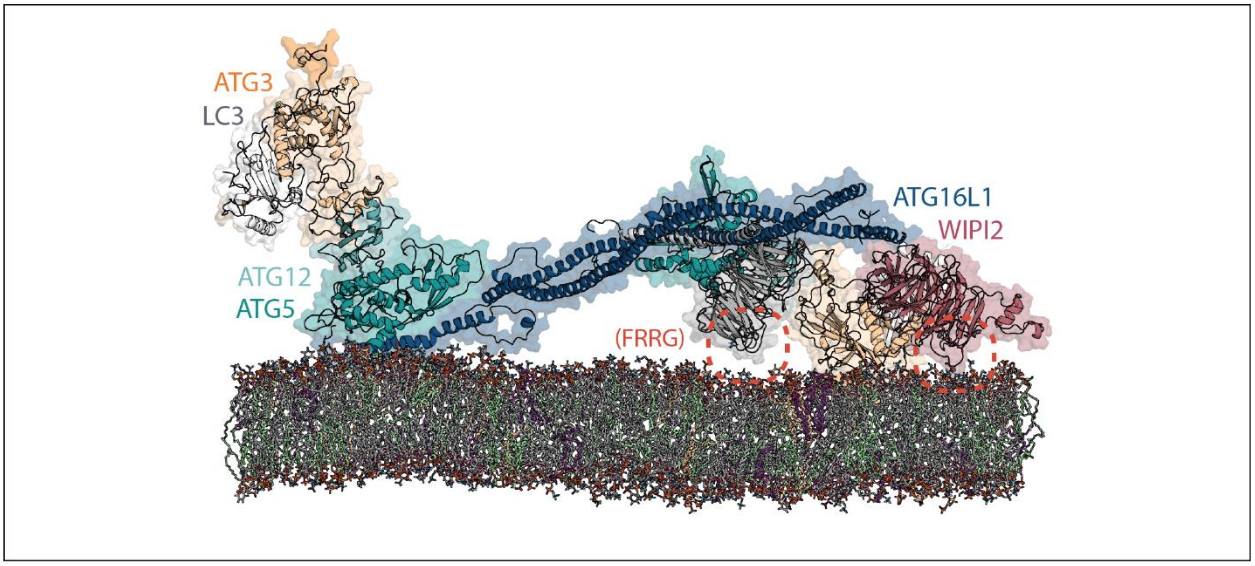

Fig. S9

Binding of a second WIPI2 to interaction site within the ATG16L1 coiled coil is compatible with membrane-engaged configuration of ATG12–ATG5-ATG16L1 complex from atomistic molecular dynamics simulation. Locations of the membrane-interacting FRRG motif of the first (pink) and second (gray) WIPI2 molecule are each indicated with a red dashed oval.

Table S1.

Membrane interaction data collection from all-atom molecular dynamics simulations performed in this study. Sets of simulations with alternative local mean membrane curvature (*H*) or histidine protonation state (protonated at the imidazole δ- or ϵ-nitrogen or both, '+') are indicated.

<table>
<tr><th colspan="3">Simulated construct of complex</th><th>Number of replicates</th><th>Simulation duration (μs)</th></tr>
<tr><td colspan="3">WIPI2-ATG16L1-ATG5–ATG12-ATG3–LC3</td><td>5</td><td>1</td></tr>
<tr><td colspan="3">ATG16L1-ATG5–ATG12-ATG3–LC3</td><td>5</td><td>1</td></tr>
<tr><td colspan="2" rowspan="4">ATG16L1-ATG5–ATG12-ATG3–LC3, after membrane insertion of ATG3 helix</td><td>H ~0.065 nm^{-1}</td><td>3</td><td>1</td></tr>
<tr><td>H ~0.05 nm^{-1}</td><td>3</td><td>1</td></tr>
<tr><td>H ~0.03 nm^{-1}</td><td>3</td><td>1</td></tr>
<tr><td>H < 0.01 nm^{-1}</td><td>3</td><td>1</td></tr>
<tr><td rowspan="6">ATG3-LC3</td><td rowspan="4">His262/His266 uncharged</td><td>His262 (δ), His266 (δ)</td><td>5</td><td>2</td></tr>
<tr><td>His262 (δ), His266 (ϵ)</td><td>5</td><td>2</td></tr>
<tr><td>His262 (ϵ), His266 (δ)</td><td>5</td><td>2</td></tr>
<tr><td>His262 (ϵ), His266 (ϵ)</td><td>5</td><td>2</td></tr>
<tr><td rowspan="2">His266 doubly protonated</td><td>His262 (δ), His266 (+)</td><td>5</td><td>2</td></tr>
<tr><td>His262 (ϵ), His266 (+)</td><td>5</td><td>2</td></tr>
</table>

Movie S1

Dynamics of the membrane-recruited ATG12–ATG5-ATG16L1-WIPI2 complex loaded with the ATG3-LC3 conjugate (yellow/white) during all-atom molecular dynamics simulation. The movie corresponds to the final 550 ns of a 1 μs simulation. Water and ions in the system are not shown, for clarity.

Movie S2

Delivery of ATG3-LC3 (yellow/white) to the membrane by the ATG12–ATG5-ATG16L1 complex. Beginning from an initial configuration with a membrane-embedded helix α2 of ATG16L1 (dark blue), the ATG3-LC3 conjugate reached the membrane spontaneously, with the ATG3 N-terminal helix (orange) inserted in between membrane lipids. The movie corresponds to the first 600 ns of a 1 μs all-atom molecular dynamics simulation. Water and ions in the system are not shown, for clarity.
